# Cases of Variola and Vaccine

**Published:** 1849-11

**Authors:** George L. Upshur

**Affiliations:** Norfolk, Va.


### SELECTIONS

FROM

### AMERICAN AND FOREIGN JOURNALS.

---

*Cases of Variola and Vaccine.* By George L. Upshur, M. D., of Norfolk, Va.—Small pox prevailed in this city to a considerable extent during the first three months of this year. In the house where I saw my first case, was a mother with an infant four months old, both unprotected by vaccination. I vaccinated them with some very active virus, and on the eighth day, when the vesicle was fully formed, I directed them both to be carried into the room where the patient with small pox was lying, then the eighth day from the appearance of the eruption. From that day, both mother and child were freely exposed to the infection, and both escaped without the slightest indisposition. The grandfather of the infant, who had been inoculated thirty years before, and had the variolous disease fully, as shown by marks upon his person, had varioloid severely, as did five of his children who had had varioloid some years before, not one in the family escaping the disease except the grandmother and the mother and child before mentioned. This would seem to prove that *fresh* vaccination is a better prophylactic than *old* small pox.

My second case of variola occurred in the person of a negro, who was seized with a chill on Sunday, and broke out on the following Wednesday. I saw her for the first time on Friday; the eruption was then just passing from the papular into the vesicular stage. Her child, three years old, had been sleeping with her from the beginning of her sickness. I immediately vaccinated it upon the left arm in two places. Ten days after, and when the mother was convalescing, there was no trace of the punctures upon the child's arm. I vaccinated her again in the right arm. A week after this the child was seized with all the symptoms of small-pox. Upon examining the arms, I found that upon the *left* one were two vaccine vesicles. The case turned out to be one of very mild varioloid.

I saw another case, in the practice of Dr. Tunstall, which had been very freely exposed to the variolous infection, and in whose system the vaccine virus lingered for fourteen days without manifesting itself locally. It at length made its appearance simultaneously with the variolous disease, and seemed so to modify it as to make the case one of mild varioloid.

I treated all the cases I had under my care with a purgative, now and then, of calomel and rhubarb, and a solution of nitrate of potassa during the high febrile stage. The fact frequently mentioned in the books, particularly struck me, viz: that the initiatory symptoms of varioloid are fully as severe as those of confluent small pox.

Since the 15th of January, I have vaccinated 179 persons, of which 40 were *re*-vaccinations. Of these last, 34, or about 75 per cent., took the disease a second time. The susceptibility in some cases to the disease, a second and even a third time, is singular. In 1846 I vaccinated a little girl, with success—in '47 repeated the operation with the same result—in '48 vaccinated her again, and she again took the disease. Last spring, upon trying it again, no impression was produced even locally. She has a brother and a sister, who have each had the vaccine disease perfectly, twice. The scars from the last operation are just as distinct and characteristic as from the first.—*Med. Examiner, Oct.*, 1849.

